# Comparative Study of Dexamethasone-Loaded Thermoresponsive In Situ Gels and Polymeric Micelles for Ocular Drug Delivery

**DOI:** 10.3390/ijms26178414

**Published:** 2025-08-29

**Authors:** Boglárka Szalai, Orsolya Jójárt-Laczkovich, Anita Kovács, Szilvia Berkó, Bence Sipos, Gábor Katona, Mária Budai-Szűcs

**Affiliations:** Institute of Pharmaceutical Technology and Regulatory Affairs, Faculty of Pharmacy, University of Szeged, Eötvös Street 6, H-6720 Szeged, Hungary; szalai.boglarka@szte.hu (B.S.); jojartne.laczkovich.orsolya@szte.hu (O.J.-L.); gasparne.kovacs.anita@szte.hu (A.K.); berko.szilvia@szte.hu (S.B.); sipos.bence@szte.hu (B.S.); katona.gabor@szte.hu (G.K.)

**Keywords:** corneal PAMPA, ex vivo, in situ gel, ocular drug delivery, permeation, poloxamer, polymeric micelle

## Abstract

Effective ocular drug delivery is still a challenge for pharmaceutical technologists due to the complex elimination mechanisms of the eye. In situ gels and polymeric micelles are among the pharmaceutical technologies that may enable us to overcome these challenges. Therefore, the objective of this study was to evaluate the ocular applicability of in situ gels and polymeric micelles, as well as their combinations, containing a steroidal anti-inflammatory drug, dexamethasone. The developed formulations were compared on the basis of their physicochemical characteristics, rheological behavior, mucoadhesion, in vitro drug release profile, and in vitro and ex vivo permeability. The developed formulations exhibited moderate stability according to the zeta potential measurements; however, they demonstrated appropriate mucoadhesion and sustained drug release. Furthermore, the results of the permeability studies suggest that combining thermoresponsive in situ gels and polymeric micelles represents a promising strategy for enhancing the therapeutic efficacy of ocular drug delivery.

## 1. Introduction

Among corticosteroids, dexamethasone (DXM) is considered one of the most potent and efficient anti-inflammatory drugs for the treatment of diseases of the anterior and posterior segments of the eye [1,2]. As corticosteroids generally, DXM has poor water solubility (0.16 mg/mL at 25 °C [3]); therefore, in topical formulations, DXM is typically administered as a suspension [2]. However, the bioavailability of these formulations is less than 5% because of the ocular barriers and rapid clearance [4,5]. Despite the potential for enhancing the water solubility of DXM through the use of its more soluble salt form, a recent study demonstrated that the weak acid form (dexamethasone) exhibited superior corneal permeation when compared to the ionized form (dexamethasone sodium phosphate). Moreover, corticosteroids with low solubility are the pharmaceutical agents of choice for polymeric slow-release delivery systems [2]. To overcome the challenges of effective drug delivery, DXM is available as an intravitreal injection [6] and implant [7], as well as an intracanalicular insert [8]. Although a longer duration of action is provided by these pharmaceutical forms, the application requires qualified staff and may be uncomfortable, hindering patient compliance. Therefore, there is a clinical demand for topical formulations with prolonged drug release and enhanced bioavailability.

Polymer-based in situ gels have emerged as a promising approach for ophthalmic applications. These systems are liquids that, upon reaching the treatment area, undergo a phase transition to form a gel structure as a result of a physiological change [9]. Gelation can be triggered by pH, temperature, and ions, as well as external factors, such as UV radiation [9,10]. Among these systems, thermoresponsive in situ gels are one of the most studied, with some commercially available eye drops containing polymers, for example, hydroxypropyl methylcellulose, poloxamer 407, and polycarbophil [9]. In situ gels based on P407 have the potential to enhance residence time on the eye surface and to provide controlled and prolonged drug release [11,12].

Generally, poloxamer 407 is an amphiphilic triblock copolymer composed of poly (ethylene oxide) (PEO) and poly(propylene oxide) (PPO), from which biocompatible and transparent gels can be prepared [12]. Gelation occurs due to hydrophobic interactions between PPO blocks as the temperature increases, resulting in micelle formation and gelation [13,14]. Therefore, the gelling temperature can be precisely adjusted by the concentration of P407 [13]. Also, the gelation is reversible, which is vital for these drug delivery systems. In addition, poloxamer 407 can enhance the ocular bioavailability of drugs, as it is a surfactant [15]. It may also enhance permeation by increasing the solubility of drugs, removing phospholipids from the epithelial cell membrane but not damaging it, and relaxing the epithelium cell junction [15].

Furthermore, poloxamer 407 is also known for its solubilizing capacity [16]. However, the amount of it applied in the formulations of this study was not sufficient to solubilize the ocular therapeutic dose of DXM (1 mg/mL). Therefore, a β-cyclodextrin derivative was used to solubilize DXM. The optimal derivative and amount were determined in our previous study [17]. Cyclodextrins (CD) are cyclic oligosaccharides composed of 6–8 glucopyranose units [18]. They are shaped like truncated cones with hydrophilic outer surfaces and hydrophobic cavities [19]. Poorly water-soluble molecules or parts of them can fit into these cavities, thus forming inclusion complexes with CDs [19]. CDs can not only increase water solubility, but also enhance the ocular absorption of drugs (e.g., dexamethasone, pilocarpine, and cyclosporins) and reduce local irritation [20]; therefore, their application in ocular dosage forms can be advantageous. Furthermore, poloxamer 407 is an excipient widely used in the pharmaceutical industry that is generally recognized as safe (GRAS) [21]. In a recent study, nanoemulsion-based in situ gels containing 13% poloxamer 407 were formulated and systematically evaluated for ocular safety. The optimized composition was tested using cell viability assessment (MTT assay), the hen’s egg test on chorioallantoic membrane (HET-CAM), and the Draize ocular irritation test. The results showed that the formulation was non-toxic to human retinal pigment epithelial cells, caused no irritation or vascular damage, and was well-tolerated [22]. Collectively, these findings provide evidence for the ocular safety of poloxamer 407 at the concentration used in this study.

Another approach to improving ocular bioavailability is polymeric micelles. Polymeric micelles are nanoscale structures formed by the self-assembly of amphiphilic block copolymers in aqueous media. These structures generally consist of a hydrophilic outer shell and a hydrophobic core that enable the encapsulation of hydrophobic drugs. Among polymeric micelles, a polyvinyl caprolactam–polyvinyl acetate–polyethylene glycol graft copolymer (Soluplus) has attracted great interest due to its exceptional solubilization capability and the capacity to enhance the bioavailability of poorly water-soluble compounds [23,24]. Soluplus-based micelles have been demonstrated to enhance drug permeation primarily through the transcytosis of intact micelles across the corneal epithelium. During this process, micelles are internalized by endocytosis and subsequently released via exocytosis at the basolateral surface, allowing the drug to reach deeper ocular layers. Despite the absence of a comprehensive understanding of the precise mechanism, studies have demonstrated that Soluplus nanomicelles may significantly enhance ocular permeation via the topical route [25]. Moreover, Soluplus has demonstrated ocular safety in both in vitro and in vivo studies. In a formulation containing 10 mg/mL Soluplus and 25 mg/mL poloxamer 407, the HET-CAM assay showed no signs of irritation [26]. Similarly, another study using 0.4 mM Soluplus reported no hemorrhage, vascular lysis, or coagulation in the HET-CAM test, confirming its non-irritant profile [25]. In vivo evaluation in albino rabbits further supported its safety, as a Soluplus-based polymeric micelle formulation exhibited a zero irritation index, indicating excellent ocular tolerance [27]. Together, these results confirm the suitability of Soluplus for ophthalmic applications.

A review of extant literature reveals numerous studies addressing the ophthalmic use of in situ gels [28,29,30,31] and polymeric micelles [32,33,34,35], as well as the combination of the two systems [36,37,38,39], yet comparative studies with the same active ingredient are seldom reported. The objective of the present study is to compare the ocular absorption of dexamethasone-containing polymeric micelles and thermoresponsive in situ gels, as well as to evaluate the combination of these systems using in vitro and ex vivo ophthalmic models. The findings of this study may contribute to the optimization of ophthalmic drug formulations, with a particular focus on increasing the efficacy of ocular anti-inflammatory therapy.

## 2. Results

### 2.1. Physicochemical Characterization

Particle size and polydispersity index (PDI) are critical parameters in nanocarrier formulations. In this study, sample SP and sample P407 are classified as nanocarriers, since the particle size range between 10 and 100 nm corresponds to nanoparticles [40]. Furthermore, the size distribution of SP polymeric micelles was ideally low, with a PDI value below 0.2, which is considered acceptable for polymeric nanoparticles [41]. In contrast, the particle size of the hybrid systems was substantially larger, which was concomitant with a higher PDI value (Table 1). The particle size and PDI measurements were conducted at ambient temperature, which may have initiated the slight aggregation of the poloxamer micelles, leading to an increase in the particle size and a broadening of the size distribution. In the case of the SP + P407 (r) sample, the particle size increased to more than 10,000 nm, which was beyond the application range of the DLS technique.

The zeta potential, which is defined as the electrokinetic potential on the surface of a particle, is a critical factor that determines the stability of the particles [42]. The SP polymeric micelle and the P407 in situ gel exhibited moderate stability with zeta potential values of −7.72 mV and −11.02 mV, respectively. However, the hybrid system SP + P407 had a neutral or a near-neutral charge.

The EE% values of the formulations were all above 80%, which is favorable for efficient therapeutic drug delivery.

### 2.2. Rheological Study

Gelling temperature is a pivotal characteristic of thermoresponsive in situ gels as they are applied as a free-flowing liquid and gelling occurs due to body heat. Therefore, the gelling temperature should be above room temperature but below the corneal temperature (34–35 °C [43]).

Thermal gelling of SP was not expected, as no poloxamer was incorporated into this sample. There is a clear reverse correlation between the poloxamer concentration and the gelling temperature; with increasing the concentration of poloxamer 407, the gelling temperature decreases [44]. Based on our previous work, a concentration of 12% *w*/*w* poloxamer 407 was chosen as it resulted in optimal gelation [17] with a gelling temperature of 33.2 ± 0.69 °C (sample P407). Although the poloxamer reconstituted SP + P407 (r) sample contained the same concentration of poloxamer as sample P407, gelation occurred at a higher temperature (38.8 ± 0.18 °C). Furthermore, when SP and P407 were co-lyophilized (SP + P407 (r)), no gelling was observed (Figure S1).

### 2.3. Mucoadhesion Study

Mucoadhesion plays a vital role in ocular drug delivery by increasing the residence time on the ocular surface, thus enhancing bioavailability. Poloxamer 407 is known to have mucoadhesive properties [45].

**Figure 1:**
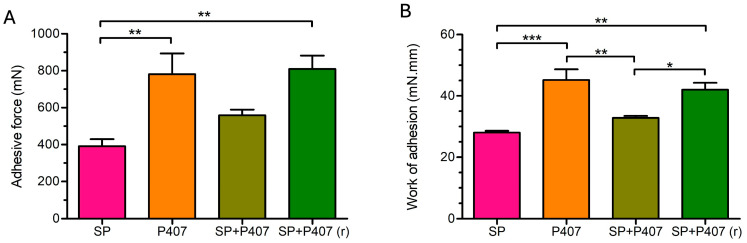
Mucoadhesion properties of the samples (**A**) adhesive force; (**B**) work of adhesion. Statistical analysis: ANOVA followed by Tukey’s multiple comparison test. * *p* ≤ 0.05 significant; ** *p* ≤ 0.01 very significant; *** *p* ≤ 0.001 highly significant difference.

Regarding both adhesive force and work of adhesion, P407 and SP + P407 exhibited equivalent mucoadhesion. Furthermore, they showed significantly higher mucoadhesion compared to SP and SP + P407 (r) (Figure 1). The difference in mucoadhesion between SP + P407 and SP + P407 (r) may be attributed to the assumption that SP + P407 does not form a gel structure, as evidenced by the rheological studies. The combination of the two polymers, Soluplus and poloxamer 407 increased mucoadhesion compared to SP; however, the difference between SP and SP + P407 was not statistically significant.

### 2.4. In Vitro Drug Release Study

**Figure 2:**
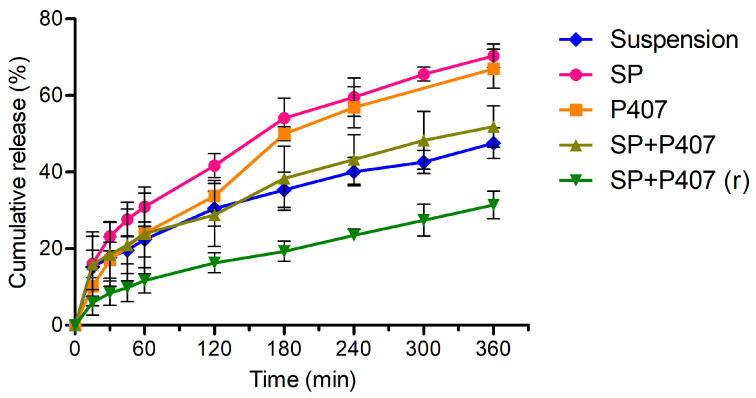
Cumulative release of DXM from different dosage forms: suspension, polymeric micelles, in situ gels, and hybrid systems.

The cumulative release curves of the formulations are shown in Figure 2. The release curves of SP and P407, P407 and SP + P407, as well as the suspension and SP + P407, and the suspension and P407 were similar with f2 similarity factors of 63.674, 52.860, 72.896, and 53.041, respectively. In contrast, the other pairs of samples showed no similarity. SP, P407, SP + P407, and the suspension had a relatively fast initial drug release with a cumulative drug release of 31.0%, 24.1%, 24.0%, and 28.2% at 60 min, respectively. After the first 60 min of the study, drug release slowed down but still showed a constant release. On the contrary, SP + P407 (r) demonstrated a low but constant drug release rate throughout the 360 min of the test. In order for DXM to be released from the SP + P407 (r) formulation, it is first necessary for the DXM to be released from the polymeric micelles. Subsequently, the DXM must diffuse through the gel matrix, resulting in a steady drug release with a cumulative release lower than that of the suspension. In the case of the DXM suspension, a limited amount of solvent penetrates the dialysis membrane, facilitating the dissolution of DXM, while a certain amount of DXM is already present in the dissolved form. The release rate is therefore determined by the combined effect of the dissolution of DXM and the diffusion of the dissolved drug across the membrane. Despite the absence of gel formation, SP + P407 demonstrated a lower cumulative release compared to SP and P407. This phenomenon can be explained by the release of DXM from two polymeric systems: the micelles and the surrounding poloxamer 407 layer, even in the absence of a gel structure. The combination of these two polymers resulted in a decrease in the rate of drug release. This combination hindered drug release to a lesser extent when no gel structure was formed (i.e., SP + P407) compared to SP + P407 (r). These results suggest that a combination of polymeric micelles and in situ gels provide a sustained release of DXM, especially when Soluplus-based polymeric micelles are reconstituted in a poloxamer 407 solution. In this case, micro-sized aggregates may have formed and slowed down the diffusion of DXM. Sustained drug release, together with increased mucoadhesion, is advantageous for enhancing ocular bioavailability of drugs, reducing dosing frequency, and improving overall efficacy of the treatment.

Model fitting was also performed to provide a more detailed interpretation of the drug release. Among the zero-order, first-order, Higuchi, and Korsmeyer–Peppas models, the Korsmeyer–Peppas model presented the best fit for all samples (Table 2). This model was designed to describe the drug release from polymeric systems. In this model, drug release can be classified according to the *n* value. In the present study, the samples were placed in tube-shaped membranes; therefore, the geometry was cylindrical. SP and SP + P407 exhibited Fickian diffusion, suggesting that the driving mechanism of drug release was diffusion. On the other hand, gel forming samples (SP + P407 (r) and P407) presented *n* values suggesting non-Fickian (anomalous) transport. In this case, drug release was driven by diffusion and swelling. The anomalous effects were caused by the slow rearrangement of the polymeric chains of poloxamer 407 and the concurrent diffusion [46]. In the case of the DXM suspension, the release kinetic was driven by the dissolution rate of the DXM particles, which resulted in a lower *n* value.

### 2.5. In Vitro Corneal-PAMPA

The Corneal Parallel Artificial Membrane Permeability Assay (Corneal-PAMPA) is an innovative in vitro model designed to predict the permeability of drugs through the cornea. The cornea is composed of hydrophilic and lipophilic layers. Corneal-PAMPA models the composition of the epithelium, the layer that most influences absorption through the cornea [47], without accounting for the hydrophilic nature of the stroma. Consequently, highly lipophilic compounds may exhibit high permeability in the corneal-PAMPA; however, under in vivo or ex vivo conditions, their diffusion through the hydrophilic stroma may be significantly slower or even negligible.

**Figure 3:**
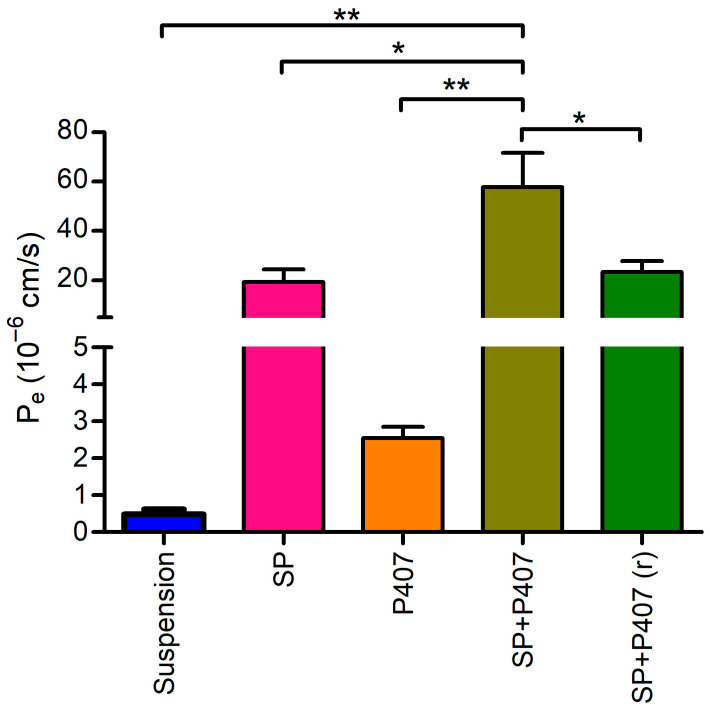
Comparison of the effective permeability (P_e_) of different formulations containing dexamethasone. Statistical analysis: ANOVA followed by Tukey’s multiple comparison test. * *p* ≤ 0.05 significant; ** *p* ≤ 0.01 very significant difference.

For this assay, the dilution of the formulations was not required. The formulations containing Soluplus exhibited substantial effective permeability (P_e_) values (Figure 3). Conversely, the DXM suspension demonstrated low permeability, likely attributed to the slow dissolution of the suspended particles. Poloxamer 407 has been observed to increase permeability [48]; however, only a slight increase in permeability was observed for sample P407 compared to the suspension. It is hypothesized that this phenomenon may be attributed to the inclusion complex formed with the cyclodextrin, as only the free fraction of DXM can cross biological membranes. In the absence of the cyclodextrin, encapsulating DXM into polymeric micelles significantly increased permeation, particularly in the context of the co-lyophilized hybrid system (SP + P407). In this case, Soluplus and poloxamer 407 enhanced permeability through the lipophilic membrane in a synergistic manner. When Soluplus-based polymeric micelles were reconstituted in the poloxamer 407 solution (SP + P407 (r)), permeability did not increase compared to SP. This phenomenon may be explained by the formation of micro-sized precipitates, which have been shown to slow down the release of DXM. These precipitates may also adhere to the membrane, thus limiting the permeability.

Membrane retention was determined for the calculation of the effective permeability of DXM. The membrane retention of SP, P407, SP + P407, and SP + P407 (r) was 57.9%, 22.3%, 58.0%, and 67.0%, respectively. This result indicated that the SP-containing samples may integrate or adhere to the lipophilic membrane, increasing the accumulation of DXM within the membrane. Furthermore, micro-precipitates of the SP + P407 (r) sample presented the highest membrane retention, which resulted in less DXM in the acceptor phase, thus lowering the permeability, as can be observed in Figure 3.

### 2.6. Ex Vivo Porcine Eye Study

**Figure 4:**
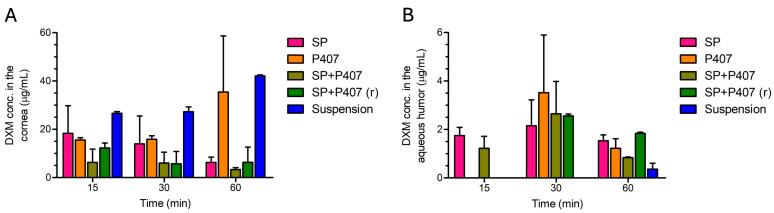
DXM concentrations measured (**A**) in the cornea and (**B**) in the aqueous humor after treatment with different formulations containing DXM.

In the ex vivo permeability study, whole porcine eyes were utilized for the measurement of the DXM content in two compartments. The evaluation was conducted on the cornea and aqueous humor (Figure 4). However, it is important to acknowledge the possibility that DXM might have permeated other parts of the eye, such as the posterior segment, which were not examined in this study. Quantification of these tissues requires further development of the model, a topic not addressed in this study.

The results demonstrate that within the initial 15 min of study, the SP polymeric micelle and the P407 in situ gel exhibited the highest concentrations of DXM in the cornea, with the exception of the suspension. The SP sample also showed promising permeability as indicated by the PAMPA model. Samples containing Soluplus exhibited the capacity to yield sufficient amounts of DXM in both the cornea and the aqueous humor within 15 min. Conversely, lower concentrations were observed in the cornea after treatment with the hybrid systems.

After 30 min, the differences observed in the 15 min measurements were diminished, with all the polymeric micelles, the in situ gel, and the hybrid systems yielding substantial quantities of permeated DXM in both compartments examined. The highest concentration at 30 min was observed after treatment with P407, which contained DXM in a dissolved state, in the form of CD inclusion complexes.

Subsequently, at the 60 min measurement, the concentration of DXM generally decreased in the cornea and the aqueous humor, suggesting that the drug may have migrated towards the posterior segment of the eye, thereby reducing the concentration in the anterior segment.

The reference DXM suspension showed outstanding corneal penetration, which can be attributed to its dissolution in the saline solution applied in the precorneal space. The free dissolved DXM molecules could easily penetrate the cornea, resulting in high corneal concentrations. It is evident that DXM accumulated in the cornea; however, its presence in the aqueous humor was observed only after a 60 min treatment period, with minimal amounts detected in the aqueous humor. This finding indicates that not only the polymeric micellar system and the poloxamer-based gel but also their combinations exerted a favorable influence on the deeper permeation of DXM and on achieving a more substantial DXM in the postcorneal space.

## 3. Discussion

In this study, we developed and compared dexamethasone-loaded thermosensitive in situ gels, polymeric micelles, and hybrid systems for ophthalmic applications. The formulations were evaluated in terms of physicochemical properties, rheological behavior, mucoadhesion, in vitro drug release, and permeability characteristics using both in vitro and ex vivo models. The aim was to assess the potential of these systems to improve drug retention and ocular bioavailability.

Polymeric micelles have the potential to form nanocarriers with a monodisperse particle size distribution as evidenced by sample SP (D_H_ = 69.46 nm, PDI = 0.13). However, combining Soluplus and poloxamer 407 resulted in an increase in particle size and PDI (SP + P407: D_H_ = 584.10 nm, PDI = 0.56; SP + P407 (r): D_H_ > 10,000 nm) to such an extent that these hybrid systems can no longer be considered nanosized. A recent study also showed that poloxamer-based mixed micelles had a particle size over 200 nm after drug loading [49].

Zeta potential values above |30| mV suggest total electrostatic stabilization, whereas limited flocculation occurs between |5| and |15| mV [50]. The zeta potential of the in situ gel (P407: −11.02 mV) and the polymeric micelle (SP: −7.72 mV) fell in the range of limited flocculation. Although a higher absolute value of the zeta potential is required for optimal stability, Abdulkarim et al. demonstrated that neutral or near-neutral particles exhibit enhanced diffusion through mucus membranes [51]. This aspect of the near-neutral hybrid system (SP + P407: −0.0251 mV) is particularly relevant for the rapid and effective ocular delivery of drugs. For micelles prepared from Soluplus, poloxamer 407 and (2-Hydroxypropyl)-β-cyclodextrin (HPBCD), Klahan et al. also demonstrated slightly negative zeta potential values and a particle size of approximately 60 nm [52]. Given the limited stability of the formulations in this study, it is recommended that the samples be stored in lyophilized form and subsequently dissolved immediately prior to use.

In all formulations, the solubilization of DXM was achieved by HPBCD or the polymers. This solubilization resulted in the presence of DXM in a molecularly dispersed distribution, accompanied by favorable drug encapsulation efficiency (more than 80%), as demonstrated in Table 1.

Regarding the rheological study, P407, SP + P407, and SP + P407 (r) contained equivalent amounts of poloxamer 407; yet significant differences in gelation were observed. The P407 sample exhibited an optimal gelling temperature of 33.2 °C. However, the incorporation of polymeric micelles into the system resulted in an increase in the gelling temperature to a point where gelation could no longer occur at body temperature (38.8 °C). A recent study demonstrated that some polymers can strongly increase the gelling temperature of poloxamer 407 [53]. Furthermore, as poloxamer 407 can form micelles, it has a capability to form mixed micellar systems as well [14,54,55], which may significantly influence gelation. A remarkable finding was that although SP + P407 and SP + P407 (r) were the same except for the preparation method, there was a pronounced difference in their rheological properties, with the complete absence of gelation in the case of SP + P407. The co-lyophilization of the two polymers inhibited the gel-forming capacity of poloxamer 407.

In the permeability studies, the hybrid systems demonstrated remarkable potential for enhancing the ocular bioavailability of DXM; therefore, further optimization of the gelling properties is recommended. For SP + P407 (r), the gelling temperature could be reduced by increasing the poloxamer concentration. In the case of SP + P407, it is hypothesized that a portion of the poloxamer could be co-lyophilized with Soluplus, while the remaining portion could be utilized for the reconstitution of the micelles.

Regarding mucoadhesion, formulations capable of forming a gel structure (SP + P407 (r) and P407) demonstrated the highest adhesive force and work of adhesion values. The mucoadhesion of the formulations is adequate; however, it could be further enhanced by incorporating even minimal amounts of mucoadhesive polymers, such as chitosan, hydroxypropyl methylcellulose, sodium or zinc hyaluronate, and Carbopol [3,56,57,58].

The drug release of the formulations was predominantly bi-phasic, characterized by a rapid initial phase followed by a sustained release phase. This characteristic is particularly beneficial for ocular drug delivery as it has the potential to quickly achieve the minimal effective concentration after administration and then maintain it during the sustained release phase [59]. Polymeric micelles without a gel structure (SP and SP + P407) exhibited Fickian diffusion; however, the formation of a gel structure modified the release mechanism to an extent that it turned into a non-Fickian diffusion (SP + P407 (r) and P407). Although in the case of some formulations a macroscopic gel structure did not form, the gelation mechanism of poloxamer 407 is known to involve the initial formation of polymeric micelles, followed by their packing into a three-dimensional gel network upon further temperature increase [60]. It is therefore reasonable to assume that SP + P407 formed a micellar system similar to the SP formulation. The presence of these polymeric micelles can act as a dispersed polymeric network that controls drug release, which justifies the applicability of the Korsmeyer–Peppas model to describe the release of dexamethasone from these systems.

As for permeability, Soluplus-based polymeric micelles can significantly enhance permeability through lipophilic membranes compared to conventional suspension, which is in accordance with literature [26,61,62]; however, these formulations showed a relatively high membrane retention (22.3–67.0%). As both Soluplus and poloxamer 407 form micelles that can solubilize lipophilic compounds, the high membrane retention values may be due to interactions between the polymers and the lipid membrane of the corneal-PAMPA [63]. Another theory for the explanation of the high retention is that the micelles may integrate or adhere to the lipophilic membrane. Such interactions may therefore lead to an apparent overestimation of membrane binding in the corneal-PAMPA model. In contrast, the ex vivo permeability studies conducted on isolated porcine cornea did not reveal any significant accumulation of dexamethasone within the corneal tissue. These findings indicate that, although the corneal-PAMPA model provides valuable mechanistic insights, it may not fully predict the in vivo situation regarding drug retention. Consequently, our results suggest that long-term ocular drug accumulation is unlikely to occur with the tested hybrid formulations, and the high retention observed in the corneal-PAMPA should be interpreted with caution in light of the limitations of this model. On the other hand, the ex vivo study demonstrated that polymeric micelles, along with their combination with in situ gels, may facilitate penetration of DXM into deeper tissues of the eye when compared to a conventional suspension. Furthermore, SP + P407 exhibited noteworthy permeability in both permeability studies, which results can also be attributed to the near-neutral zeta potential of this formulations [51]. This finding, together with the mucoadhesive properties of the hybrid systems, suggests that these combined formulations represent a promising novel approach to enhance ocular bioavailability of DXM. However, further optimization of the gelling temperature is necessary to ensure the optimal performance of these formulations.

This study was limited to in vitro and ex vivo evaluations; no in vivo experiments were performed to confirm the translational relevance of the findings. The corneal-PAMPA model and the ex vivo porcine eye study, while useful for preliminary screening, does not fully replicate the complexity of ocular tissues and physiological conditions. Additionally, only one corticosteroid, dexamethasone, was tested, and the results may not be directly extrapolated to other drugs with different physicochemical properties. Finally, the long-term stability and ocular safety of the formulations were not experimentally addressed in this work and should be investigated in future studies.

Generally, scaling up these formulations for clinical use will require optimization of manufacturing methods to ensure reproducibility, sterility, stability, and regulatory compliance. Preclinical safety and efficacy studies, followed by clinical trials, are essential to confirm the observed benefits. Cost-effective production and GMP-compliant processes will be key for successful commercialization.

## 4. Materials and Methods

### 4.1. Materials

Dexamethasone (DXM) was obtained from Pharmacia and Upjohn Company LLC (Kalamazoo, MI, USA). (2-Hydroxypropyl)-β-cyclodextrin (DS~4.5; HPBCD) was purchased from Cyclolab R&D Ltd. (Budapest, Hungary). Kolliphor^®^ P407 (oxyethylene 71.5–74.9%; P407) was provided by Sigma-Aldrich (St. Louis, MO, USA). Soluplus^®^ (polyvinyl caprolactam–polyvinyl acetate–polyethylene glycol graft copolymer; SP; average molecular weight ≈ 118,000 g/mol, nominal range 90,000–140,000 g/mol) was kindly gifted by BASF GmbH (Hannover, Germany). D-trehalose dihydrate (TRE), mucin from porcine stomach, components of phosphate-buffered saline (pH = 7.4; PBS), and components of the simulated tear fluid (pH = 7.4; STF) were purchased from Sigma-Aldrich Co. Ltd. (Budapest, Hungary). The STF was prepared according to Hägerstöm et al. as follows: 6.78 g/L NaCl, 2.18 g/L NaHCO_3_, 0.084 g/L CaCl_2_⋅2H_2_O, and 1.38 g/L KCl were dissolved in purified water, and pH was adjusted with 0.1 M HCl [64]. tert-Butanol (t-ButOH) was obtained from Molar Chemicals LLC (Halásztelek, Hungary). Methanol (HPLC gradient grade; MeOH) and acetonitrile (HPLC gradient grade; ACN) were provided by Chem-Lab NV (Zedelgem, Belgium).

### 4.2. Sample Preparation

Four formulations were investigated: poloxamer-based in situ gel (P407), Soluplus micelles (SP), a combined lyophilized system (SP + P407), and poloxamer-reconstituted Soluplus micelles (SP + P407 (r)). All formulations had a DXM concentration of 1 mg/mL.

The concentrations of Soluplus and poloxamer 407 were selected based on previous studies conducted in our laboratory. A phase solubility study demonstrated that a Soluplus concentration of 25–30 mg/mL substantially increased the solubility of dexamethasone [36]. A factorial design study with poloxamer 407 investigated concentrations of 12–18% (*w*/*v*), identifying 12% as optimal for ophthalmic application [17].

The in situ gel (P407) was prepared according to the modified cold method [65]. As DXM is poorly soluble in water (0.16 mg/mL [3]), 9.44 mM HPBCD was applied in this formulation to solubilize DXM determined in our previous work [17]. Therefore, the first step of sample preparation was the complete dissolution of HPBCD and DXM in purified water facilitated by sonication. Then, 12% *w/w* P407 was added to the solution, and after thorough mixing, the formulation was left at 4 °C overnight to let the polymer dissolve. Finally, the formulation was homogenized.

Polymeric micelles were prepared as follows: a polymeric solution of 30 mg/mL of Soluplus and 12% *w/w* of P407 was prepared in purified water. P407 was incorporated into the system at this step only in the case of the sample SP + P407. A DXM solution of 1 mg/mL was prepared in t-ButOH. Then, equal volumes of the polymeric and DXM solutions were mixed, and TRE was added to the mixture as a cryoprotectant, resulting in a TRE concentration of 5% *w/v*. The mixture was stirred for 4 h at 750 rpm at room temperature. Subsequently, volumes of 1.5 mL of the mixtures were transferred into freeze-drying vials and first, the samples were frozen at −40 °C, then freeze-dried using a ScanVac CoolSafe 100–9 (LaboGene Aps, Lynge, Denmark) equipment (−40 °C, 0.013 mbar, 12 h). After freeze-drying, a secondary drying was performed at 25 °C and 0.013 mbar for 6 h. The freeze-dried cakes were stored at 4 °C. For the evaluation of the samples, the freeze-dried cakes were dissolved in 1.5 mL of purified water (SP and SP + P407) or were reconstituted in 1.5 mL of 12% *w/w* P407 solution (SP + P407 (r)).

A DXM suspension was also prepared using purified water as the reference system. The particle size of the suspension was 6.2 µm.

### 4.3. Measurement of Particle Size, Size Distribution, and Zeta Potential

The particle size (D_H_ = average hydrodynamic diameter) and the particle size distribution (PDI = polydispersity index) of the formulations were measured by a Malvern Nano ZS Zetasizer (Malvern Instruments, Malvern, UK) based on the dynamic light scattering (DLS) method. The samples were loaded into folded capillary cells, and measurements were carried out at 25 °C. The refractive index was considered 1.592. The same apparatus was used for the measurement of the zeta potential (ζ) of the formulations.

### 4.4. Encapsulation Efficiency

The encapsulation efficiency (EE%) was determined using the indirect method. Briefly, 1 mL of the formulation was transferred into Vivaspin 15R 5000 MWCO Hydrosart tubes (Sartorius, Stonehouse, UK). The aqueous media containing the free fraction of DXM was separated via centrifugation (Hermle Z323 K high-performance refrigerated centrifuge, Hermle AG, Gosheim, Germany) at 10,000 rpm and at 4 °C for 30 min.

The DXM content of the filtered solution was measured using an HPLC method described in Section 4.7. The following equation was used to calculate the EE% (Equation (1)):

(1) $$EE\%=\frac{Initial\;DXM\left(mg\right)-Measured\;DXM(mg)}{Initial\;DXM(mg)}\times 100$$

### 4.5. Rheological Study

The gelling temperature of the samples was measured by a Physica MCR 302 Modular Compact Rheometer (Anton Paar, Graz, Austria) using a cone and plate type measuring device of a diameter of 25 mm, a cone angle of 1°, and a gap height of 0.05 mm. The measurement was performed at a constant frequency (1 rad/min) and strain (1%) from 15 °C to 40 °C at a heating rate of 1 °C/min. The crossover of the storage modulus (G′) and the loss modulus (G″) curves was considered the gelling temperature. The tests were performed in triplicates.

### 4.6. Mucoadhesion Study

Mucoadhesion tests were conducted using a TA.XT plus Texture Analyzer (Stable Micro Systems Ltd., Surrey, UK) that was equipped with a 5 kg load cell. A filter paper was saturated with 50 μL of 8% *w/w* mucin dispersion [66], which was prepared with STF, and then the filter paper was positioned within the mucoadhesion test rig. The test rig and the mucosal surface were heated to 35 °C, which is approximately the physiological corneal temperature. This temperature was maintained during the tests. Subsequently, 20 mg of each sample was applied to the cylinder probe, which had a diameter of 10 mm. Initially, the sample and the mucosal surface were subjected to a preload of 2500 mN for 3 min. Thereafter, the probe was elevated at a rate of 2.5 mm/min to disrupt the adhesive bond. To determine the adhesive force and work of adhesion values of the formulations, five parallel measurements were performed [67].

### 4.7. In Vitro Drug Release Study

The dialysis bag method was applied for the investigation of the in vitro drug release of the formulations. Briefly, approximately 600 mL of the samples were loaded into cellulose dialysis membrane tubes (Zellutrans/Roth, Carl Roth GmbH + Co. KG, Karlsruhe, Germany, MWCO: 12,000–14,000 Da), which were 10 mm in width and 6.37 mm in diameter. The tubes were then closed using Spectra/Por^®^ closures (Spectrum Laboratories Inc., Rancho Dominguez, CA, USA) and weighed to obtain the precise weight for the calculations. Subsequently, the filled dialysis bags were placed in 25 mL of STF heated to 35 °C. The release medium was continuously stirred using the magnetic stirrer to ensure homogeneity. The sink condition was maintained during the course of the study. At the sampling times (15, 30, 45, 60, 120, 180, 240, 300, and 360 min), 1 mL of the release medium was collected, then immediately replaced with 1 mL of fresh STF. For each formulation, 3 parallel measurements were carried out. The drug release of the in situ gel, the polymeric micelles and the combined systems was also compared with that of the DXM suspension and the solution of the inclusion complex.

To further assess the drug release of the samples, model fitting and bootstrap f2 analysis were employed using an Excel add-in, DDSolver [68]. The f2 similarity value is a critical metric in comparing drug release curves, with values between 50 and 100 being considered similar [69].

The concentration of DXM was measured using an HPLC system (Shimadzu Nexera X2 UHPLC, Kyoto, Japan) with a C18 reverse-phase column (Phenomenex Kinetex C18, 2.6 μm, 100 Å, 150 × 4.6 mm, Phenomenex, Torrance, CA, USA). The separation process was conducted using a gradient elution method according to the following program: time (min)/% of CAN: 0/35, 4.0/60, 4.01/35, 6.0/35. For each sample, 5 μL were subjected to analysis at a wavelength of 240 nm. The measurements were conducted at a flow rate of 1 mL/min and utilizing a column temperature of 40 °C. The analysis time and the retention time of DXM were 6 min and 3.2 min, respectively.

### 4.8. In Vitro Corneal-PAMPA

The in vitro transcorneal permeability of the formulations was investigated by means of the corneal parallel artificial membrane permeability assay (PAMPA) [47]. Corneal-PAMPA is a non-cellular, high-throughput assay intended for early screening of passive corneal permeability and membrane retention [70]. The artificial lipid membrane and the absence of biological processes, such as tear turnover and active transporters, may limit direct in vivo extrapolation. Reported datasets show weak correlation between corneal-PAMPA and Caco-2 permeability values indication independence from this model [71]. Furthermore, multivariate quantitative structure-property relationship models trained on larger corneal-PAMPA sets demonstrated good predictive value for the corneal permeability of pharmaceutical drugs [70]. Accordingly, the corneal-PAMPA results were interpreted alongside ex vivo porcine cornea data.

Briefly, the wells of the acceptor plate (Multiscreen Acceptor Plate, MSSACCEPTOR, Millipore, Bedford, MA, USA) were filled with 300 μL of phosphate-buffered saline (PBS, pH 7.4). Then, 16 mg of phosphatidylcholine was dissolved in a solvent mixture (70% *v/v* hexane, 25% *v/v* dodecane, and 5% *v/v* chloroform) and each well of the donor plate (MultiscreenTM-IP, MAIPN4510, pore size: 0.45 mm, Millipore, Bedford, MA, USA) was coated with 5 μL of the lipid solution. After the solvent mixture evaporated, a phosphatidylcholine lipid membrane of 10.67% *w/v* was formed. Subsequently, 150 μL of the formulations was transferred onto the lipid membrane. The permeability study was carried out at 35 °C for 4 h. Six parallel measurements were performed for each formulation.

The DXM content of the donor and acceptor phase was measured using the HPLC method described in Section 4.7.

The effective permeability (P_e_; cm/s) of DXM was calculated according to Avdeef [72] using the following equation (Equation (2)):

(2) $$P_{e}=\frac{-2.303\times V_{D}}{A\times \left(t-\tau_{SS}\right)}\times \frac{1}{1+r_{v}}\times \log\left[{-r}_{v}+\frac{1+r_{v}}{1-R_{M}}\times \frac{C_{D}\left(t\right)}{C_{D}\left(0\right)}\right]$$

where V_D_ is the volume of the donor compartment (0.15 cm^3^), A is the area of the lipid membrane (0.24 cm^2^), t is the incubation time (14,400 s), τ*_SS_* is the time to reach the steady-state (s), r_v_ is the volume ratio of the aqueous compartment (r_v_ = V_D_/V_A_, where V_A_ is the volume of the acceptor compartment (0.3 cm^3^)), R_M_ is the membrane retention factor, and C_D_(0) and C_D_(t) are the concentration of DXM (mol/cm^3^) in the donor compartment initially and after 4 h, respectively.

The membrane retention factor (R_M_) was calculated as previously described by Avdeef [73] (Equation (3)):

(3) $$R_{M}=1-\frac{C_{D}\left(t\right)}{C_{D}\left(0\right)}-\frac{V_{A}\times C_{A}\left(t\right)}{V_{D}\times C_{D}\left(0\right)}$$

where C_D_(0) and C_D_(t) are the concentration of DXM (mol/cm^3^) in the donor compartment initially and after 4 h, respectively, V_A_ is the volume of the acceptor compartment (0.3 cm^3^), V_D_ is the volume of the donor compartment (0.15 cm^3^), and C_A_(t) is the concentration of DXM (mol/cm^3^) in the acceptor compartment after 4 h. Data used for the calculations are presented in the supplementary materials (Table S1).

### 4.9. Ex Vivo Porcine Eye Permeability Study

For the ex vivo porcine eye permeability study of the DXM-containing formulations, freshly donated porcine eyes were obtained from a slaughterhouse. The eyeballs were placed in polytetrafluoroethylene cells, leaving only the cornea uncovered. The eyeballs were kept at 35 °C and treated with 900 μL of physiological saline solution and 100 μL of the formulations for 15, 30, and 60 min. For each composition, 3 parallel studies were performed.

Subsequent of the treatment, the aqueous humor was aspirated by corneal paracentesis and the cornea was excised. The aqueous humor was then diluted with an equal volume of methanol and centrifuged at 4 °C for 30 min at 16,500 rpm (Hermle Z323 K high-performance refrigerated centrifuge, Hermle AG, Gosheim, Germany), and the DXM content of the clear supernatant was measured by HPLC (Section 4.7). From the excised corneas, DXM was extracted with 2 mL of methanol:water 50:50 (*v/v*) using a PSU10i Orbital Shaker (Grant Instruments Ltd., Cambs, UK) at 450 rpm for 1 h. The extract was then filtered with FilterBio PVDF-L syringe filters (0.45 μm, Lab-ex Ltd., Budapest, Hungary). The quantitative analysis of the filtered extracts was also performed by the HPLC method described in Section 4.7. The measured DXM concentrations of the compartments are presented in the supplementary materials (Table S2).

### 4.10. Statistical Analysis

Data are presented as mean ± standard deviation (SD). Values are compared using analysis of variance (ANOVA) followed by Tukey’s test using GraphPad Prism 5.0 software (GraphPad Software Inc., San Diego, CA, USA). Differences were considered statistically significant at *p* < 0.05.

## 5. Conclusions

In this study, in situ gel and polymeric micelle-based ophthalmic formulations of DXM were compared in terms of their physicochemical properties, mucoadhesion, in vitro drug release, and in vitro and ex vivo permeability. The hybrid systems exhibited a combination of characteristics from both approaches, resulting in advantageous mucoadhesive properties, drug release kinetics, and permeability. In summary, the present findings underscore the potential of combined polymer-based drug delivery systems to enhance the ocular bioavailability of poorly water-soluble corticosteroids, such as dexamethasone. Further in vivo studies are necessary to confirm the therapeutic potential of the proposed formulations.

## Figures and Tables

**Table 1:** Average hydrodynamic diameter (D_H_), polydispersity index (PDI), zeta potential (ζ) values, and encapsulation efficiency (EE%) of the in situ gel, the polymeric micelles, and the hybrid systems. Data are presented as average ± SD (*n* = 3).

Sample	D_H_ (nm)	PDI	ζ (mV)	EE%
SP	69.46 ± 5.05	0.13 ± 0.05	−7.72 ± 0.48	88.34%
P407	33.05 ± 3.91	0.44 ± 0.13	−11.02 ± 1.27	87.74%
SP + P407	584.10 ±43.19	0.56 ± 0.04	−0.0251 ± 0.0308	83.32%
SP + P407 (r)	>10,000	n.a.	n.a.	87.84%

n.a.: not applicable.

**Table 2:** Model fitting of the in vitro drug release curves.

	Zero Order		First Order		Higuchi		Korsmeyer–Peppas		
	K ± SD	r^2^	K ± SD	r^2^	K ± SD	r^2^	K ± SD	*n*	r^2^
SP	0.24 ± 0.01	0.6704	0.0042 ± 0.0005	0.8855	3.85 ±0.248	0.9861	4.92 ± 1.756	0.46	0.9948
P407	0.22 ± 0.01	0.8175	0.0036 ± 0.0001	0.9415	3.49 ± 0.083	0.9704	2.61 ± 1.309	0.58	0.9860
SP + P407	0.17 ± 0.03	0.5418	0.0026 ± 0.0007	0.7207	2.82 ± 0.542	0.9282	5.08 ± 5.293	0.45	0.9877
SP + P407 (r)	0.10 ± 0.01	0.7762	0.0012 ± 0.0002	0.8269	1.56 ± 0.199	0.9623	1.48 ± 1.368	0.56	0.9904
Suspension	10.5 ± 1.2	0.3426	0.15 ± 0.03	0.5657	21.3 ± 3.1	0.9236	25.0 ± 5.5	0.36	0.9945

## Data Availability

Data will be made available upon request.

## Supplementary Materials

The following supporting information can be downloaded at: https://www.mdpi.com/article/10.3390/ijms26178414/s1.

## Abbreviations

The following abbreviations are used in this manuscript:

ACN	Acetonitrile
CD	Cyclodextrin
DLS	Dynamic Light Scattering
DS	Degree of Substitution
DXM	Dexamethasone
EE%	Encapsulation Efficiency
HET-CAM	Hen’s Egg Test on Chorioallantoic Membrane
HPBCD	(2-Hydroxypropyl)-β-Cyclodextrin
HPLC	High Performance Liquid Chromatography
MeOH	Methanol
PAMPA	Parallel Artificial Membrane Permeability Assay
PBS	Phosphate-Buffered Saline
PDI	Polydispersity Index
PEO	poly(ethylene oxide)
PPO	poly(propylene oxide)
SD	Standard Deviation
STF	Simulated Tear Fluid
t-ButOH	tert-Butanol
TRE	D-Trehalose Dihydrate
