# Supplementary material for: Comparative Study of Dexamethasone-Loaded Thermoresponsive In Situ Gels and Polymeric Micelles for Ocular Drug Delivery
Source: Int J Mol Sci. 2025 Aug 29;26(17):8414. doi: 10.3390/ijms26178414 (PMC12428703; doi:10.3390/ijms26178414)
Supplement: Supplementary file 1 [file ijms-26-08414-s001.zip › ijms-3813714-supplementary.pdf]

Supplementary

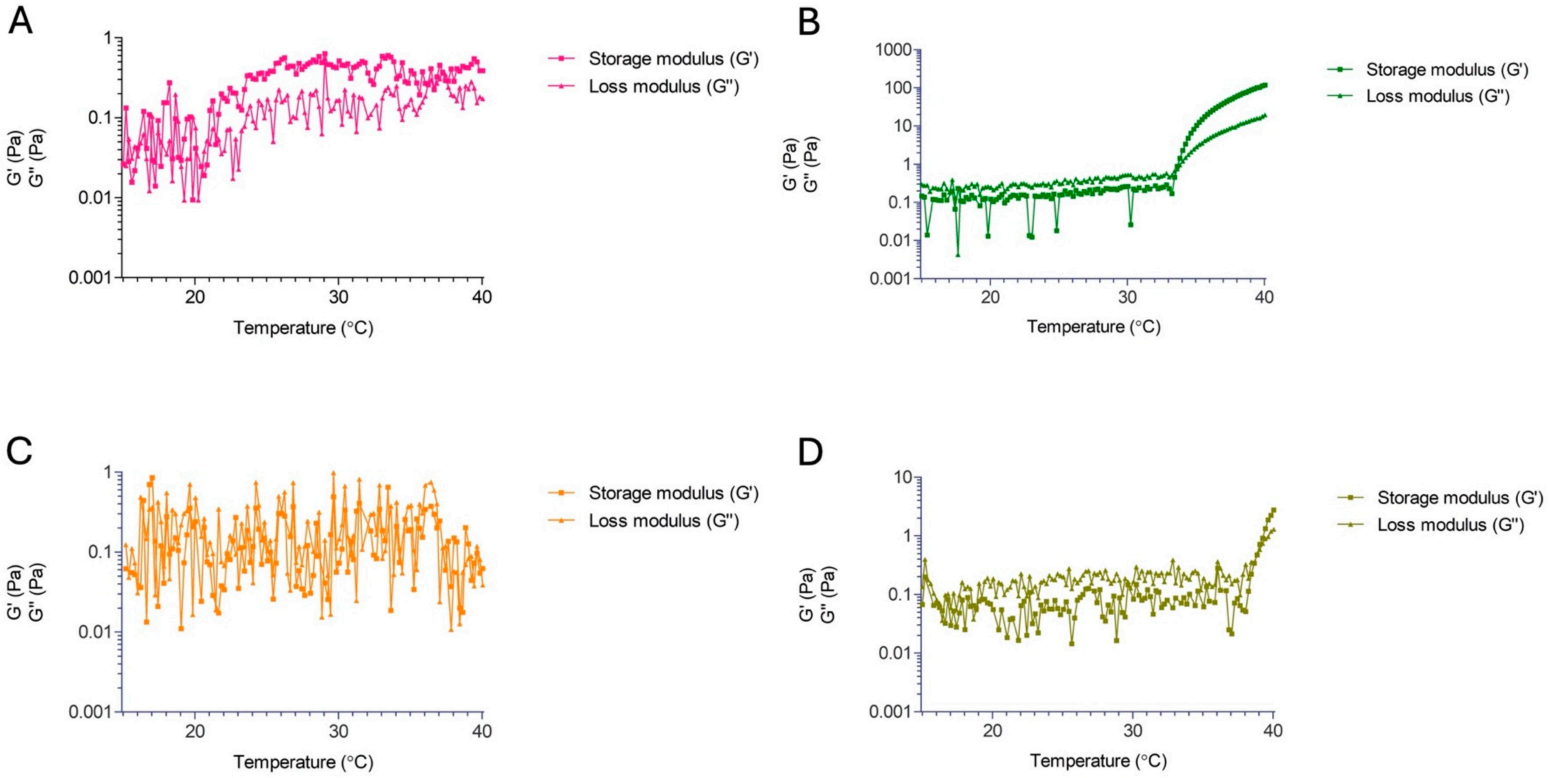

Figure 1. Gelling temperature measurements of (A) SP, (B) P407, (C) SP+P407, and (D) SP+P407 (r). The crossover of the storage modulus (G') and loss modulus (G'') curves is considered the gelling point.

Table 1. DXM concentrations measured in the acceptor and donor phase of the corneal-PAMPA.

	DXM concentration (μM) Acceptor phase						DXM concentration (μM) Donor phase					
SP	190.00	170.53	149.94	158.98	110.06	154.33	-	1123.73	764.42	341.19	1125.71	526.85
P407	133.11	162.62	110.17	114.80	117.85	102.17	320.96	305.61	346.75	311.78	303.40	381.58
SP+P407	188.32	260.84	326.94	240.42	211.00	245.13	1831.98	471.47	396.99	294.23	253.73	239.33
SP+P407 (r)	208.46	71.80	148.68	154.72	152.99	146.88	830.45	472.42	-	729.26	348.22	362.36
Suspension	3.19	4.22	7.85				370.71	226.00	441.08			

Table 2. DXM concentrations measured in the residual solution on the corneal surface, in the corneal extract, and in the aqueous humor after 15, 30, and 60 minutes of treatment with different formulations.

15	Corneal surface					Cornea					Aqueous humor				
min	µg/mL	µg/mL	µg/mL	Mean	SD	µg/mL	µg/mL	µg/mL	Mean	SD	µg/mL	µg/mL	µg/mL	Mean	SD
SP	82.85986	92.81268	86.76852	87.48	5.01	26.5337209	5.20069767	23.166124	18.30	11.47	1.95424	1.35032	1.9309	1.75	0.34
P407	74.03716	60.7809	77.48432	70.77	8.82	14.5774419	15.6244961	16.4835659	15.56	0.95	-	-	-	-	-
SP+P407 12	65.78928	66.77634	74.21394	68.93	4.61	12.6258915	3.04945736	3.12682171	6.27	5.51	0.8959	0.97346	1.79236	1.22	0.50
SP+P407 12 (r)	34.64114	44.34974	54.25388	44.41	9.81	14.6170543	11.1212403	11.0403101	12.26	2.04	-	-	-	-	-
Suspension	63.762	72.9041	58.32062	65.00	7.37	26.8076744	27.1974419	25.846124	26.62	0.70	-	-	-	-	-

30	Corneal surface					Cornea					Aqueous humor				
min	µg/mL	µg/mL	µg/mL	Mean	SD	µg/mL	µg/mL	µg/mL	Mean	SD	µg/mL	µg/mL	µg/mL	Mean	SD
SP	89.4528	67.35232	88.8218	81.88	12.58	4.26852713	26.7617054	10.9496124	13.99	11.55	0.97766	3.06768	2.41578	2.15	1.07
P407	52.37736	63.13914	51.00934	55.51	6.64	15.9768217	14.3382946	17.2409302	15.85	1.46	6.08596	3.08174	1.3791	3.52	2.38
SP+P407 12	51.15654	52.8474	40.38238	48.13	6.76	3.32139535	3.5872093	11.2157364	6.04	4.48	4.12898	1.53172	2.27472	2.65	1.34
SP+P407 12 (r)	46.20372	69.61098	61.53152	59.12	11.89	11.6176744	3.10069767	2.46317829	5.73	5.11	2.64766	2.53294	2.49922	2.56	0.08
Suspension	42.71472	41.39894	40.82386	41.65	0.97	24.9658915	28.5908527	28.2931783	27.28	2.01	-	-	-	-	-

60	Corneal surface					Cornea					Aqueous humor				
min	µg/mL	µg/mL	µg/mL	Mean	SD	µg/mL	µg/mL	µg/mL	Mean	SD	µg/mL	µg/mL	µg/mL	Mean	SD
SP	-	-	94.44404	94.44	-	5.11403101	8.83472868	4.92674419	6.29	2.20	1.2491	1.6405	1.70102	1.53	0.25
P407	70.04066	49.68504	70.02216	63.25	11.75	62.1864341	19.9469767	24.1244186	35.42	23.27	0.76592	1.42212	1.47914	1.22	0.40
SP+P407 12	51.56368	28.2889	46.37714	42.08	12.22	4.11682171	2.53992248	3.32883721	3.33	0.79	0.85834	0.84434	0.81646	0.84	0.02
SP+P407 12 (r)	33.3114	-	43.40846	38.36	7.14	1.86085271	3.67224806	13.4949612	6.34	6.26	1.8944	1.81058	1.8092	1.84	0.05
Suspension	53.39226	53.95758	57.49404	54.95	2.22	42.0168992	41.5844961	42.5631008	42.05	0.49	0.64122	0.24862	0.20074	0.36	0.24
